# Near-Ambient Pressure Oxidation of Silver in the Presence of Steps: Electrophilic Oxygen and Sulfur Impurities

**DOI:** 10.1021/acscatal.4c02985

**Published:** 2024-08-12

**Authors:** Frederik Schiller, Khadiza Ali, Anna A. Makarova, Sabine V. Auras, Fernando García-Martínez, Alaa Mohammed Idris Bakhit, Rodrigo Castrillo Bodero, Ignacio J. Villar-García, J. Enrique Ortega, Virginia Pérez-Dieste

**Affiliations:** † Centro de Física de Materiales CSIC/UPV-EHU-Materials Physics Center, San Sebastián E-20018, Spain; ‡ Donostia International Physics Center, San Sebastián E-20018, Spain; § Department of Microtechnology and Nanoscience, Chalmers University of Technology, Göteborg SE-41296, Sweden; ∥ Physikalische Chemie, Institut für Chemie und Biochemie, Freie Universität Berlin, Arnimallee 22, Berlin 14195, Germany; ⊥ Deutsches Elektronen-Synchrotron DESY, Notkestraße 865, Hamburg 22607, Germany; # ALBA Synchrotron Light Source, Cerdanyola del Vallès, Barcelona 08290, Spain; ¶ Departamento de Química y Bioquímica, Facultad de Farmacia, Universidad San Pablo-CEU, CEU Universities, Boadilla del Monte 28668, Spain; ∇ Universidad del País Vasco, Dpto. Física Aplicada I, San Sebastián E-20018, Spain

**Keywords:** silver oxidation, near ambient pressure photoemission spectroscopy, sulfur accumulation, curved crystal, catalytic substrate

## Abstract

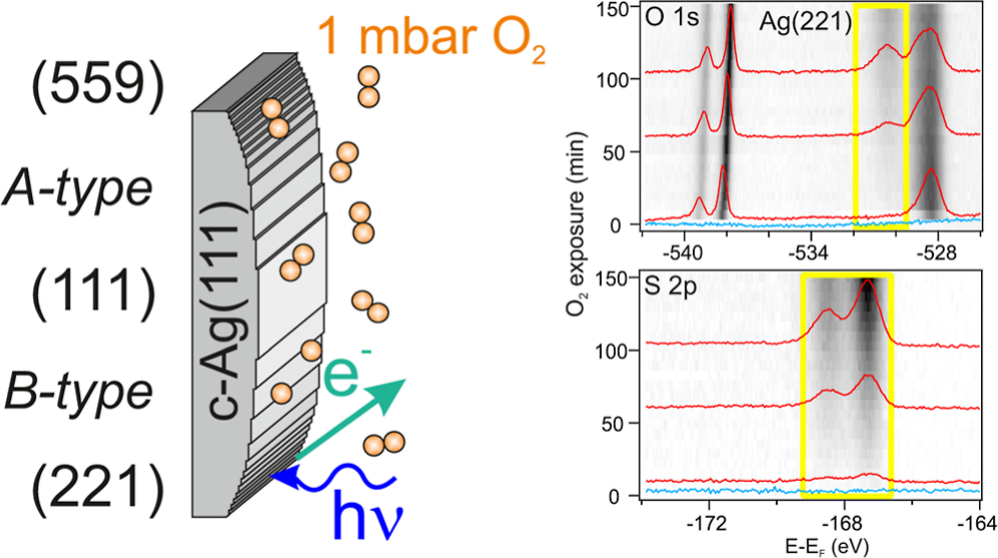

The oxidation of Ag crystal surfaces has recently triggered strong controversies around the presence of sulfur impurities that may catalyze reactions, such as the alkene epoxidations, especially the ethylene epoxidation. A fundamental challenge to achieve a clear understanding is the variety of procedures and setups involved as well as the particular history of each sample. Especially, for the often-used X-ray photoemission technique, product detection, or photoemission peak position overlap are problematic. Here we investigate the oxidation of the Ag(111) surface and its vicinal crystal planes simultaneously, using a curved crystal sample and in situ X-ray photoelectron spectroscopy at 1 mbar O_2_ near-ambient pressure conditions to further investigate surface species. The curved geometry allows a straightforward comparative analysis of the surface oxidation kinetics at different crystal facets, so as to precisely correlate the evolution of different oxygen species, namely nucleophilic and electrophilic oxygen, and the buildup of sulfur as a function of the crystal orientation. We observed that emission from both surface and bulk oxide contributes to the characteristic nucleophilic oxygen core-level peak, which arises during oxygen dosing and rapidly saturates below temperatures of 180 °C. The electrophilic oxygen peak appears later, growing at a slower but constant rate, at the expenses of the surface oxide. Electrophilic oxygen and sulfur core-levels evolve in parallel in all crystal facets, although faster and stronger at vicinal surfaces featuring B-type steps with {111} microfacets. Our study confirms the intimate connection of the electrophilic species with the formation of adsorbed SO_4_, and points to a higher catalytic activity of B-type stepped silver surfaces for alkene epoxidation or methane to formaldehyde conversion.

## Introduction

The oxidation of silver has been object of intensive investigations for nearly a century since the seminal work of Lefort, who was the first to propose silver as a metal catalyst for the synthesis of ethylene oxide.^1,2^ In fact, Ag is particularly good at promoting the epoxidation of alkenes, for example, ethen/ethylen or propene/propylene to ethylene oxide (EO)^3−6^ or propylene oxide (PO),^7^ respectively, as well as the methanol to formaldehyde conversion.^8^ EO, PO, and formaldehyde are intensively used as intermediate or final products in the chemical industry; hence, a proper knowledge of their synthesis reactions is desired. In the production process, both the epoxidation of alkenes and the synthesis of formaldehyde from methanol compete with the complete oxidation of alkenes to carbon dioxide and water. Silver-based catalysts allow the highest selectivity toward EO^5^ and formaldehyde,^8^ with nearly 90% in the EO case, yet small increases beyond that rate could have great economic and environmental impact.

During alkene epoxidation and methanol conversion to formaldehyde on silver catalysts, different silver oxides are present. The detailed characterization of these oxides and their role during the catalytic reaction, for example, whether they selectively promote the desired epoxidation/formaldehyde formation or complete alkene/methanol oxidation, is therefore a primary matter of investigation. Near-ambient pressure X-ray photoemission (NAP-XPS) has provided a lot of new information about silver oxides during the catalytic process in the case of ethylene epoxidation.^4,9,10^ At mbar pressures and within the relevant temperature range for the EO reaction (150−230 °C^9,11^), two dominant O 1s features appear in the 528−529 and 530−531 eV ranges, that is, nucleophilic and electrophilic O, respectively.^3,12−14^ The nucleophilic O would have the effect of removing one hydrogen atom from the ethylene, a process that later leads to the ethylene combustion.^5^ In contrast, the electrophilic O would attach to the C=C double bond of ethylene, forming the EO molecule. However, the very nature of electrophilic O species has been controversial over the years. It has been ascribed to oxygen atoms bound to silver either weakly^15,16^ or covalently,^17^ located at the subsurface^18^ or the surface,^19^ and has also been linked to the presence of surface carbonates,^20^ ozone^21^ or Ag_4_–O_2_ complexes.^22^ More recently, Wyrwich and Jones et al. found that the electrophilic oxygen emission was caused by trace amounts of SO_4_,^23,24^ and more importantly, they provided a strong evidence that such SO_4_ impurities would be the source of oxygen atoms in the EO synthesis.^24,25^

**Figure 1:**
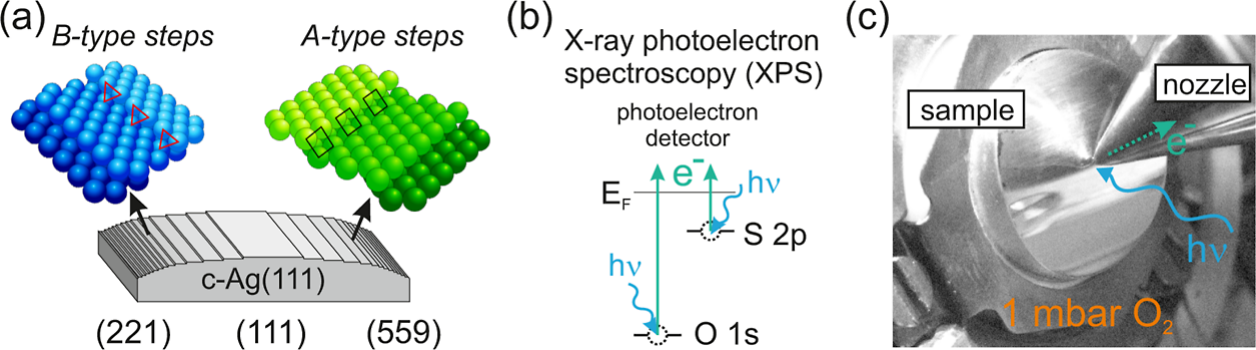
Schematic description of the curved crystal sample and NAP-XPS approach used in the present work to investigate the oxidation of Ag(111) and its vicinal planes simultaneously. (a) Curved c-Ag(111) substrate reaching all vicinal surfaces with straight steps around the vicinal angle |α| ≤ 16° of Ag(111), including A- and B-type steps between Ag(221) and Ag(559). (b) X-ray photoemission process (XPS) used during this experimental study, (c) fotograph of the sample in measurement position at the (111) surface. The nozzle defines the entrance cone to the photoelectron analyzer in the near-ambient pressure setup.

The essence of catalysis is defining and controlling active sites at the surface of the catalyst, such as atomic defects, step, and kink atoms,^26,27^ as well as foreign atoms and impurities. All of such active sites frequently coexist in industrially relevant nanoparticles, making it difficult to sort out their role in the reaction. In this context, one big challenge in surface-science-driven catalysis research is to bridge the materials gap,^28^ yet most investigations probe single crystal samples with a unique surface orientation. Therefore, a proper comparative analysis of crystal facets cannot be realized since samples are not prepared and measured under the very same experimental conditions. Instead, one can approach the multifaceted nanoparticle using “curved” crystal samples, that is, looking across the curved surface of crystal rods^29−42^ and crystal spheres,^43,44^ or at reduced cylindrical and spherical crystal sectors.^45−48^ For the latter, one can indeed match the orientation accuracy of standard single-crystal surfaces, while allowing the use of micron-size photoemission probes at its full potential,^49−53^ particularly X-ray probes in NAP-XPS.^54−56^ Here we use a cylindrical sector of a Ag single crystal to investigate the oxidation of a family of silver surfaces in the vicinity of the Ag(111) plane with XPS under near-ambient O_2_ pressure conditions. The cylindrical-sector sample is depicted in Figure 1 featuring straight steps of either A- or B-type around the Ag(111) center. We find clear spectroscopic evidence that the electrophilic oxygen is linked to the presence of sulfur oxide SO_4_ all across the curved surface. The amount of such electrophilic oxygen at different planes is very different, being maximum at surfaces featuring B-type steps. Since the amount of electrophilic O has been directly related to the epoxidation activity, we expect enhanced selectivities on such B-type vicinal surfaces toward EO production.

## Results

**Figure 2:**
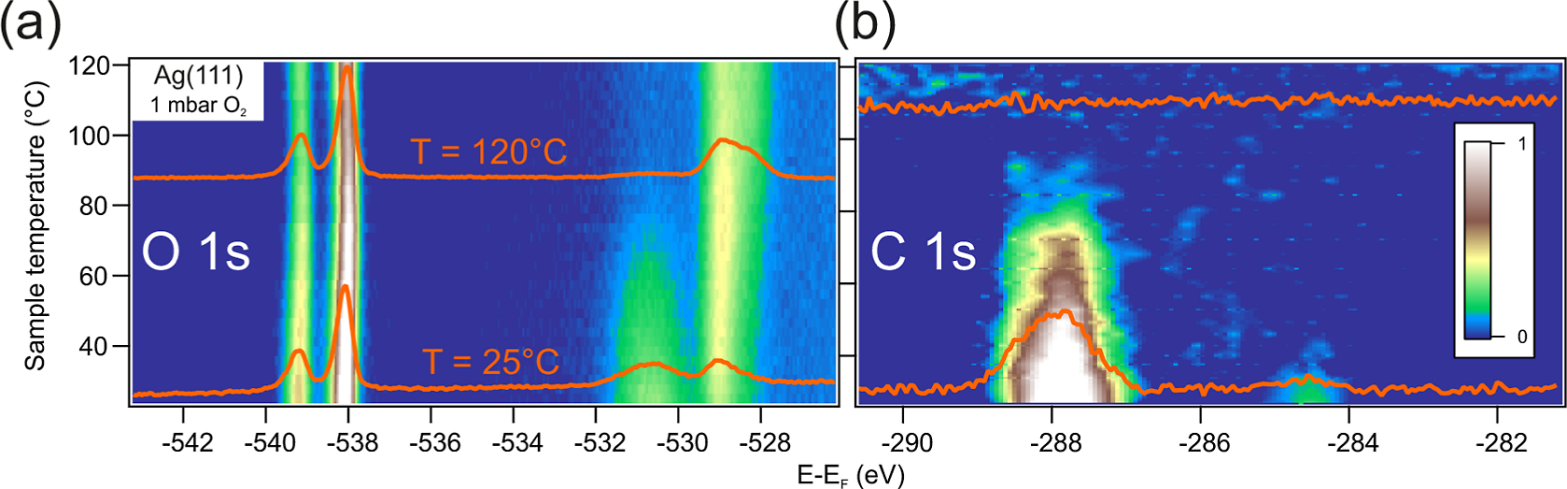
(a) O 1s and (b) C 1s temperature scans acquired at the Ag(111) surface under continuous 1 mbar O_2_ exposure, the temperature scan was started after 1 hour of exposure. The image is built with successive individual spectra, as those that appear overlaid and correspond to room temperature and 120 °C. The photon energy used was *h*ν = 620 eV, the color scale for the photoemission intensity is included as an inset. O 1s emission results from the oxygen gas doublet at high binding energies and the oxygen features from the substrate. C 1s emissions arise from surface contaminations in near-ambient pressure setups that have to be avoided for correct interpretation of the results.

**Figure 3:**
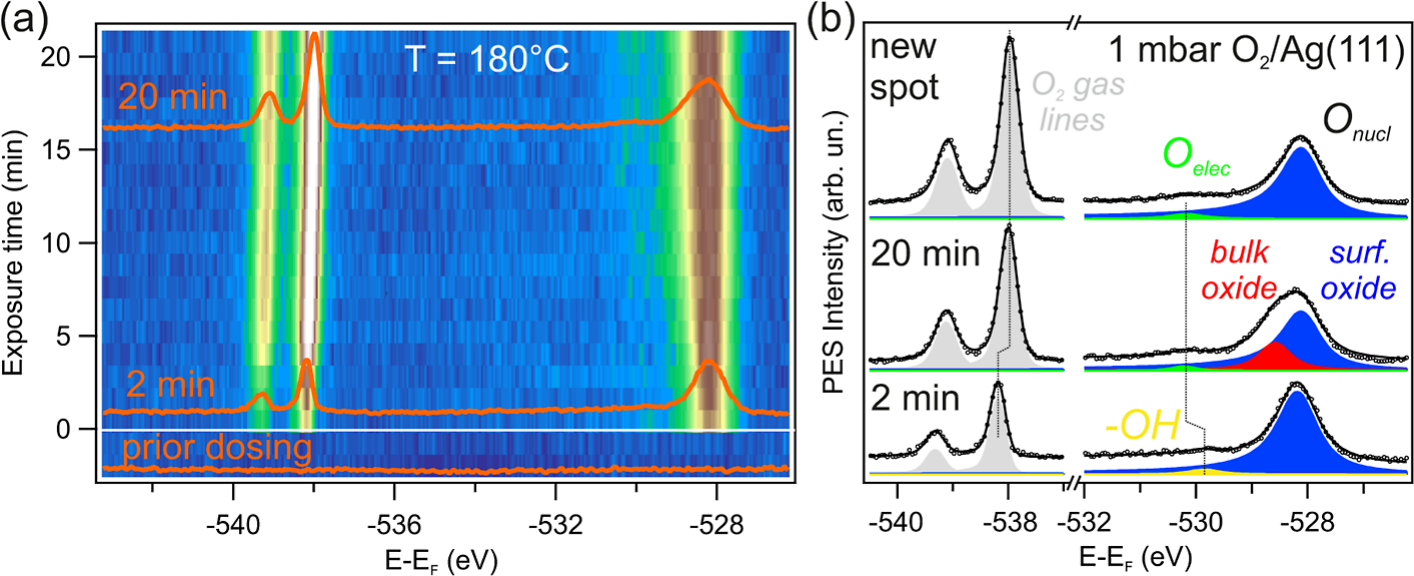
Time evolution of oxygen species at Ag(111) under 1 mbar O_2_, *T* = 180 °C. (a) O 1s image built with successive individual spectra acquired at a fixed point on the surface, showing the O_2_ gas doublet (left peak) and the surface emission (right peak). O_2_ dosing starts at *t* = 0 min. (b) Peak fit analysis to the spectra superimposed in panel (a) correspond to 2 min (bottom) and 20 min doses (center). The spectra on top are taken at a Ag(111) spot that has not been exposed to the X-ray beam during the 20 min O_2_ dosing. The absence of the subsurface/bulk oxide component in this case proves that this emission is at least partially induced by the X-ray beam.

The oxidation of silver with molecular oxygen is hard to achieve in UHV,^57^ hence atomic oxygen,^58^ ozone,^59^ or NO_2_^60^ must be used instead. In order to oxidize Ag with molecular oxygen, one requires pressures in the mbar range. However, clean environments are difficult to control in the mbar regime. On the one hand, the highest O_2_ gas quality of 6 N (99.9999%) leads to an impurity level of 10^−6^ at 1 mbar, which corresponds to roughly 1 langmuir (L) dose, that is, 1 monolayer (ML) per second. This often explains the buildup of undesired, but unavoidable contaminants that may yield misleading results.^61^ On the other hand, the interaction of the reactive gases with chamber walls, gas lines, and instruments may lead to a convective flux of organic impurities on the sample. This problem is frequent in NAP-XPS experiments performed in both, “flow” or “backfilling” mode, that is, in some silver oxidation experiments this may explain the emergence of carbon features in the spectra.^11,20,62^ Finally, to overcome photoelectron damping at high pressures, NAP-XPS is usually combined with high-brilliance, synchrotron X-ray sources, which may induce significant molecular dissociation of both O_2_ and organic impurities; that is, beam damage effects need also to be accounted for. Experiments in Figures 2 and 3 are aimed at establishing appropriate conditions for silver oxidation that minimize all such undesired effects. Other impurity and beam damage control experiments are shown in Figures S1 and S2 of the Supporting Information.

In Figure 2 we show a temperature scan of a the Ag(111) surface that has been exposed to 1 mbar O_2_ during 1 h with the curved crystal kept at room temperature (RT) and the same oxygen flow has been kept during the temperature ramp. In the O 1s spectrum we find the intense doublet at 538.1 and 539.2 eV binding energy that corresponds to oxygen gas (O_2_), whereas peaks at around 529 and 530.5 eV fall in the range of nucleophilic and electrophilic oxygen, respectively.^9^ In parallel, the C 1s spectrum exhibits a strong emission around the 288 eV binding energy and a small feature at 284−285 eV. The latter is attributed to carbidic species, whereas the former characterizes the presence of surface carbonates. These can be kept stable up to 140 °C in UHV,^3,63−67^ whereas in Figure 2 the carbidic peak disappears above 60 °C, that is, here NAP conditions appear to favor the removal of carbidic species at lower temperature. More importantly, as the temperature increases, the C 1s carbonate peak quenching in Figure 2b goes in parallel with the vanishing of the O 1s feature at 530.5 eV in Figure 2a. From this striking similarity in time evolution, we conclude that such 530.5 eV emission is due to carbonates, and that this species cannot be the active phase of the epoxidation of the alkenes, since that reaction occurs at much higher temperature, *T* ≥ 150 °C. On the other hand, the nucleophilic O 1s feature has a double peak structure at 528.2 and 528.7 eV, which is particularly well-defined at the highest temperature of 120 °C. A similar O 1s spectrum has been observed in oxidation experiments performed at UHV, where the two peaks were assigned to surface and subsurface/bulk Ag oxides, respectively.^58,68^ Of relevance for the EO formation reaction is the surface peak, which is also named as atomic oxygen or reconstruction-derived oxygen.^11,14,15,57,65^

In Figure 3 we investigate the oxygen uptake at the Ag(111) surface when exposed to 1 mbar O_2_ and constant temperature of 180 °C, in order to avoid carbonates. As indicated in Figure 3a, the O_2_ doublet and the surface oxide emission at 528.2 eV appear immediately after the valve opening (*t* = 0 min). With increasing time, one can notice a shoulder at the higher binding energy side of the surface oxide, as well as a continuous shift of the gas line features toward lower binding energies (this effect will be addressed later). The detailed peak fit analysis at valve opening (2-min exposure) and after maximum oxygen flow (20-min exposure) are presented in Figure 3b. This fit provides the energy positions of the two peaks in the nucleophilic region at 528.2 and 528.7 eV, values that are identical to those in the temperature ramp of Figure 2. At low exposure, a tiny contribution below 530 eV binding energy is detected, which appears to shift to 530.2 eV at the highest exposure. This is the binding energy range of electrophilic oxygen. After the 20 min exposure, the sample was moved along the cylinder axis to a different Ag(111) point, and a new spectrum was rapidly acquired, shown on top of Figure 3b. The emission at a binding energy of 528.7 eV is missing, but it emerges again as we keep the beam on the same spot and increase the exposure time (not shown). Therefore, the bulk oxide formation in the nucleophilic peak is at least partially beam-induced in the here used conditions.^69^ In contrast, the small emission at 530.2 eV and the O_2_ doublet are identical at both the new and the 20-min beam-exposed spots; hence, the electrophilic emission and the small gas doublet shift are not beam-induced.

**Figure 4:**
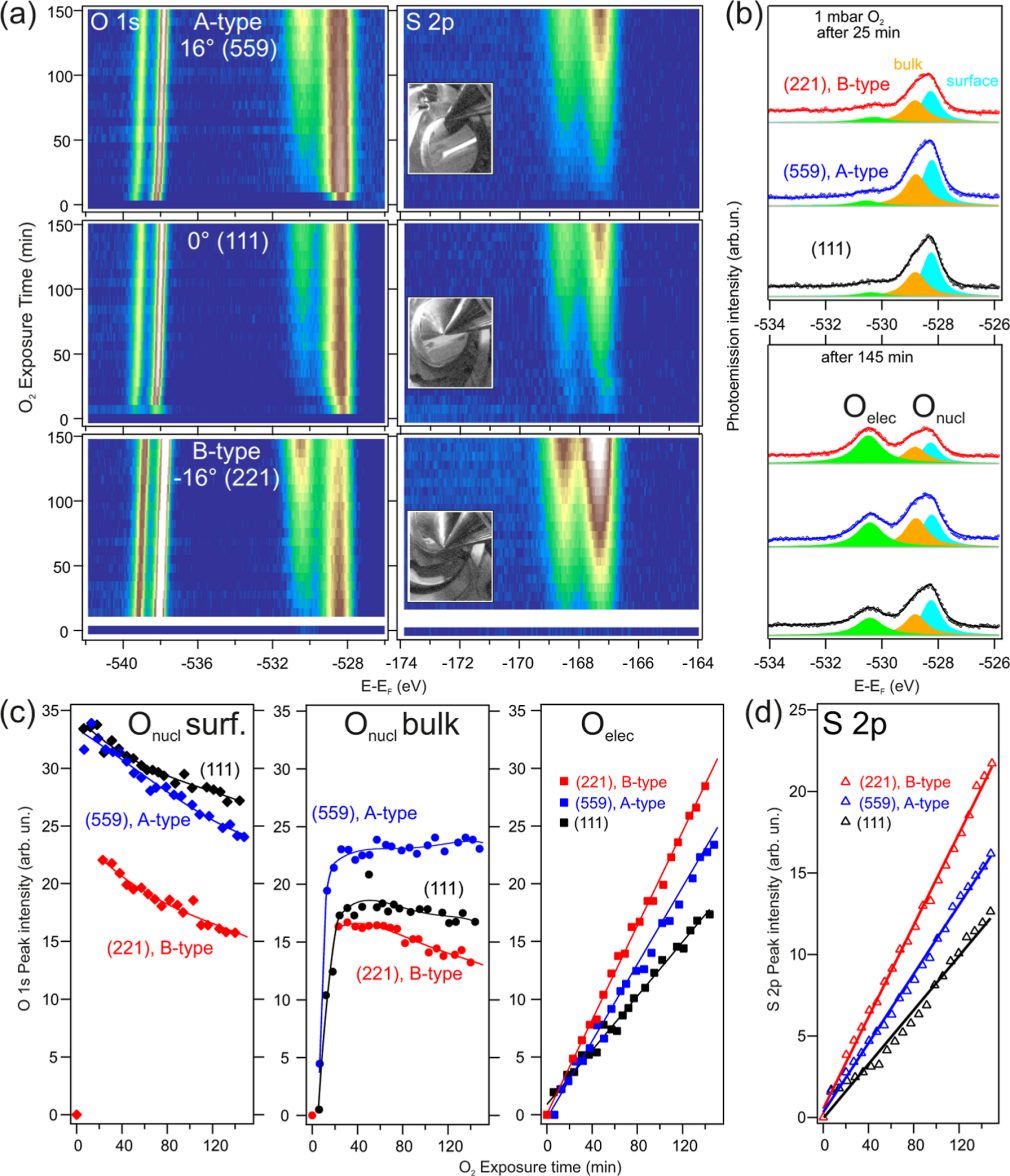
Oxygen uptake at different crystal facets on the curved Ag surface. (a) O 1s and S 2p intensity maps formed from individual spectra. The small insets are photographs of the sample/nozzle during data taking in each case. (b) Line fit analysis to individual spectra acquired after 25 and 145 min O_2_ exposure. (c) Peak intensities of the relevant nucleophilic and electrophilic oxygen components after line fitting all O 1s spectra in (a). The nucleophilic oxygen contribution is separated into surface and subsurface/bulk oxides. The lines are guide to the eyes. The decrease of the surface oxide is strong while the bulk oxide is less affected by time. (d) Integrated area of the S 2p core level in (a). For all measurements, *h*ν = 620 eV, *p*_O_2__ = 1 mbar, *T* = 170 °C.

Next, we analyze the Ag-oxidation on the vicinal Ag planes of the curved sample, i.e., at surfaces with increasing density of steps of A- and B-type (top and bottom part in Figure 1c, respectively), that is, increasing vicinal (α) angles from the (111) surface and toward the (100) and (110) surfaces, respectively. This is done here by moving the sample up and down with respect to the fixed beam spot/analyzer setup. Data shown in Figure 4 correspond to individual oxygen uptake experiments performed at three different positions over a 150 min time scale, each time on previously cleaned surfaces. This procedure is aimed at ensuring controlled conditions for direct comparison. The substrate temperature is kept constant at 170 °C and the O_2_ pressure at 1 mbar. Figure 4a displays the evolution of the O 1s and S 2p core levels for the three surfaces, namely, the sample center corresponding to the Ag(111) plane and the vicinal Ag(559) (A-type steps) and Ag(221) (B-type steps) surfaces, respectively. Some individual spectra of both, O 1s and S 2p emissions together with the Ag 3d are included in Figure S3 of the Supporting Information. The Ag 3d core level reveals only fast changes at the beginning of the oxygen exposure, and then changes are marginal. Additionally, also possible impurities are controlled, namely, C 1s and Cl 2p. The latter emissions are included in the figures of the Supporting Information, Figure S1.

As compared to Figure 3a, the longer time span in Figure 4a allows us to reveal a progressive increase of the electrophilic O 1s and the S 2p contributions in the three surfaces. In parallel, the O_2_ line doublet shows an equally uniform shift to a lower binding energy. Meanwhile, the intensity of the nucleophilic O 1s emission (surface + bulk) saturates at approximately 30 min for the three surfaces, and then it appears to decrease, particularly at the (221) plane. A more quantitative understanding can be obtained with linefit analysis to individual O 1s spectra of Figure 4a, using three peaks, i.e., the surface and subsurface/bulk oxide for the nucleophilic emission and the electrophilic O. As a way of example, Figure 4b shows the fitting results obtained after 25 and 145 min doses. Within error bars, none of the three fitting peaks reveals any binding energy change over time. Peak intensities (area under fitting lines) are plotted in Figure 4c. Notably, the surface oxide is formed at the three surfaces immediately after valve opening, whereas the nucleophilic subsurface/bulk emission needs a longer time to saturate. There is a remarkable step-dependence and A/B asymmetry in the time evolution of all species, including sulfur (Figure 4d), despite both Ag(221) and Ag(559) having the same step density (vicinal angle |α| = 16°). In fact, in the B-step type Ag(221) plane the surface oxide emission is lower than in Ag(111) and the A-step type Ag(559) surfaces by a factor of 1/3, whereas the bulk oxide decreases at the (111) and the (221) surfaces and slightly grows in the A-step (559) plane. In total, Ag(559) has the highest amount of nucleophilic (surface + bulk) Ag oxide, whereas in Ag(221) the nucleophilic oxide thickness is lower than in Ag(111). In scanning tunneling microscopy works performed on a similar curved Ag crystal, the oxygen-induced *p*(4 × 4) and *p*() superstructures were investigated, which correspond to oxygen atoms in the nucleophilic phase.^70,71^ These experiments showed that surface oxidation in B-type steps was hindered by the atomic structure. Our data presented in Figure 4 confirms these findings with respect to the nucleophilic oxygen, that is, reduced presence of surface oxide at B-type steps.

The steady buildup of electrophilic ions of S and O in Figure 4c,d shows a strikingly parallel time evolution in the three surfaces, which strongly demonstrates the direct correlation of both species at all facets. For a quantitative analysis, the atomic density of the electrophilic O with respect to sulfur has been estimated from the corresponding 1s and 2p core-level lines. This requires considering the measured instrumental sensitivity and gas-attenuation effects at the respective kinetic energies,^72^ as well as the tabulated cross sections.^73^ We obtain a S/O stoichiometry of 1:(3.8 ± 0.6), i.e., close to that of the SO_4_ compound. The parallel increase of the electrophilic oxygen and the S 2p peak together with the relation of S/O = 1:4 leads to the conclusion that in the present experiment the electrophilic peak is related to the formation of SO_4_, as proposed in ref (24). In the light of the time evolution of all species shown in Figure 4, one may qualitatively state, first, that in B-type stepped surfaces, where the formation of the surface oxide is hindered, the nonoxidized areas get rapidly covered by SO_4_, and second, that the accumulation of SO_4_ partly occurs at the expenses of both the surface (more) and the subsurface (less) nucleophilic oxides, being particularly obvious in the case of the B-stepped surface. Although suggestive, these features alone are not sufficient to demonstrate a higher reactivity and selectivity toward EO at B-type steps. However, since the surface temperature falls in the relevant range for ethylene epoxidation, our results certainly point to the presence of SO_4_ on the surface during epoxidation at 1 mbar.

**Figure 5:**
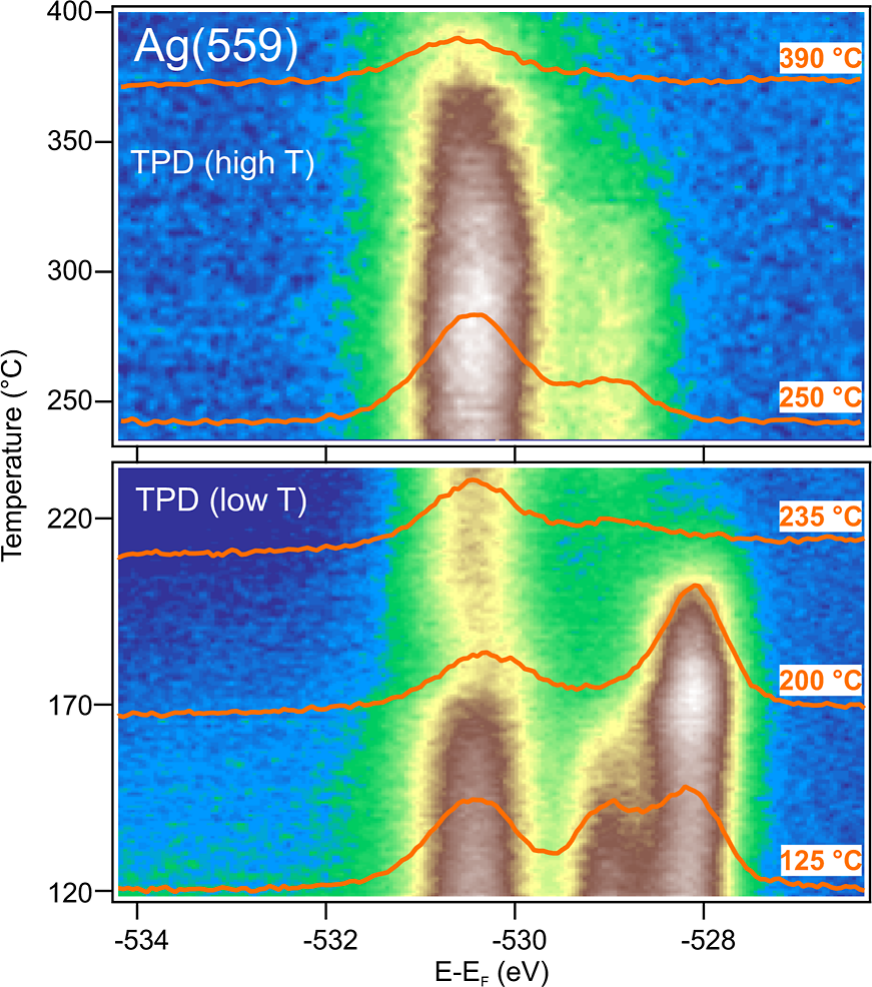
Thermal programmed desorption at Ag(559): XPS O 1s spectra during TPD experiment after 4 h of O_2_ exposure at Ag(559), *h*ν = 620 eV.

In order to check for the thermal stability of the different oxygen species, thermally programmed desorption (TPD) experiments were carried out. For this purpose, after the oxygen uptake shown in Figure 4, the O_2_ flow was stopped and the temperature was slowly increased at a constant rate while monitoring the O 1s emission. The results for the (559) surface are listed in Figure 5. The temperature was increased up to 400 °C. For better visualization, the results are split in two panels. One can observe that the bulk oxide is the less stable species. Already at 150 °C, no bulk oxygen is detected anymore. The electrophilic oxygen arising from the SO_4_ exhibits an intensity drop at 170 °C, that is, at this temperature SO_4_ may either decompose or desorb partially, or alternatively, sulfur may rediffuse into the bulk. Nevertheless, the emission does not disappear completely, only a partial drop is detected. Next, at approximately 200 °C, the surface oxide vanishes. Note that a small contribution remains at a binding energy of 531 eV, which, according to Rocha,^11^ is due to the presence of the electrophilic O_γ_ species that survives at higher temperatures during silver oxidation. Near 330 °C, the O_γ_ emission largely disappears. Nevertheless, at the highest temperature of the experiment, a small amount of the electrophilic O associated with the SO_4_ is still present at the surface. The latter observation agrees well with earlier works that found the electrophilic emission more stable than the nucleophilic one.

**Figure 6:**
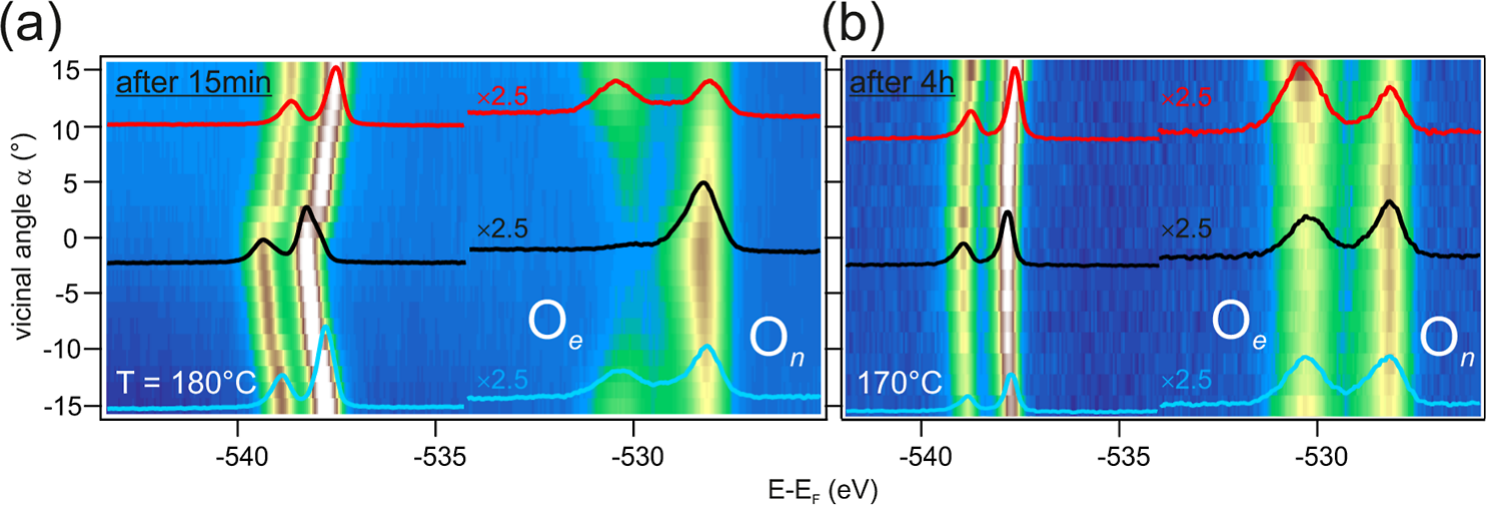
O 1s core level maps across the curved crystal. (a) Surface scan starting 15 min after valve opening, with continuous 1 mbar O_2_ flow at *T* = 180°. (b) O 1s surface map after 4 h of 1 mbar O_2_ exposure at *T* = 170 °C.

Beyond the analysis at the selected Ag(111), A-type Ag(559) and B-type Ag(221) crystal orientations, cylindrical samples allow to reveal very detailed step-density-dependent trends through the so-called α-scans, in which individual spectra are acquired point-by-point over the curved surface, such as to elaborate a map of surface species.^74,75^ Quality α-scans take long measuring time, hence steady–state reactions are required,^54−56,76,77^ although very controlled experimental conditions may be sufficient.^78−81^ In a dynamically changing scenario like the one considered here with a continuous SO_4_ increase, such α-scans are challenging. Here, we manage to achieve reasonably stable conditions during fast O 1s and S 2p α-scans (17 sample positions, 3 min duration of measurement at each position). O 1s spectra shown in Figure 6 are acquired at two different oxidation stages, that is, 15 min after valve opening, reflecting the initial stage, and after 4 h of O_2_ dosing corresponding to the stage of higher SO_4_ accumulation. The corresponding S 2p scans are displayed in the Supporting Information, Figure S5. To account for the time variations of species during data taking, reference spectra at three positions are acquired at the end of the α-scan, see Figure S6. These spectra exhibited a minor shift of the O_2_ gas feature, negligible changes in bulk/surface nucleophilic oxides, and a small increase (30%) of the electrophilic oxygen signal from the beginning to the end of the scan, especially for the first measured point in the α-scan. Therefore, the two α-scans in Figure 6 are qualitatively meaningful, i.e., they mirror the step-density dependence of all oxygen species at quite different O_2_ doses. In Figure 6a, after only 15 min exposure, nucleophilic oxygen is largest at the (111) center compared to the stepped edges, while electrophilic oxygen is lowest. This illustrates a clear inverse trend in the distribution of nucleophilic and electrophilic oxygen across the stepped surface. It suggests that the growth of electrophilic species occurs at the expense of nucleophilic oxygen. However, in Figure 6b, after prolonged exposure, this stark contrast in oxygen species distribution on the stepped surface diminishes as we approach a more stable state. This smoothing effect arises from the system having adequate time to uniformly enrich all surfaces during extended exposure. Another possibility is that the kinetics of the surface oxides is different. This may include limited Ag–O or SO_4_ diffusion on the vicinal surfaces. Nevertheless, on both images it is obvious that electrophilic O, that is, SO_4_, grows faster and thicker as the step density increases, specially at the B-type (positive α). On the other hand, the oxygen gas lines present a rightward shift in both step directions. Such a shift is caused by a change in the local work function, which perfectly correlates with the amount of SO_4_ on the surface, as discussed in the Supporting Information Figure S8.

The question that remains is the origin of the sulfur “contamination” during the oxidation process. Previous works on ethylene epoxidation pointed either to trace amounts of sulfur in the ethylene gas or to the silver crystal itself.^24^ Here, no ethylene gas has been used, although sulfur may also originate in the oxygen gas source. Even the highest purity oxygen commercially available (see methods section) may have sulfur containing gases (e.g., SO_2_, H_2_S, or OCS) in the subppm or ppb level, as these gases are present in the atmosphere. On the other side, sulfur contamination of silver is a well-known phenomenon, often described as tarnishing of Ag.^82,83^ Such tarnishing happens under ambient conditions and gets accelerated at certain temperatures and humidities.^84^ Suggestions exists on how to effectively remove tarnished layers, mostly by abrasive methods.^85^ The latter means that sulfur contamination is at the micrometer level after several centuries of ambient exposure but proves that S may travel through the Ag surface in shorter time. This implies that any exposure of a silver object to ambient conditions leads with time to an accumulation of S in the near surface region. In the case of Ag nanoparticles, structural isomer evolution with air exposure time has been observed.^86^ To check the sulfur contamination effect on the crystals in the here considered case, we used two different curved samples. In one sample the sulfur emission increased faster than in the other one. Moreover, samples that were kept under vacuum for a long time, having undergone several cycles of oxidation or ethylene epoxidation, were less prone to form electrophilic O. In Figure S7 of the Supporting Information, we include a comparison of a α-scan on two different samples to reveal such an effect. We conclude that, in a significant amount, sulfur segregates from bulk Ag. One should take into account that the sulfur content here appears under very well controlled laboratory conditions with the purest available oxygen gas and highest single crystal purity. In industrial catalytic processes involving silver nanoparticles and less pure gas feeds, the presence of S on the surface may be expected to increase considerably.

## Conclusions

We have performed a systematic analysis of the oxidation of vicinal silver surfaces under 1 mbar O_2_, using a curved Ag crystal. The surface oxide is immediately formed, and with a small time delay, subsurface/bulk oxide growth takes place, making up together the nucleophilic O 1s emission in the XPS spectra. Yet, the growth of the bulk oxide, which saturates after 30 min, appears to be at least partially beam-induced. Electrophilic oxygen at 530.2 eV binding energy is observed to linearly increase with time, in parallel to the sulfur S 2p emission at 167.3 eV binding energy, characteristic for the SO_4_ oxide. Such correlation, plus the absence of other impurities, allows one to assign the electrophilic oxygen emission to SO_4_ solely. The necessary sulfur for this formations segregates at least partially from the bulk of the Ag substrate.

The amount of electrophilic oxygen is highest at surfaces with B-type steps featuring {111}-like microfacets, compared to the A-type stepped surface ({100}-like microfacets) and the high-symmetry Ag(111) plane. At the same time, the nucleophilic oxygen is lowest at the B-type steps. During the exposure of the sample to O_2_, it seems that the electrophilic oxygen displaces the surface oxide of the nucleophilic emission, while the subsurface/bulk oxide remains nearly constant. This effect is again stronger at the B-type steps. It is very important to point out that the amount of SO_4_ at the B-type Ag(221) doubles compared to the Ag(111) surface, and it is also higher as compared to the A-type Ag(559). This will probably influence the rate of the epoxidation reaction, especially ethylene epoxidation, which would be higher and more selective at B-type stepped surfaces.

## Methods

The near-ambient pressure photoemission experiments were performed at the CIRCE-NAPP branch^87,88^ of BL24 of the ALBA synchrotron in Barcelona, Spain. The available energy range at the beamline is 90−2000 eV and the beam spot size is 100 × 20 (H × V) μm^2^. The overall energy resolution in experiment conditions (pass energy of 10 eV, exit slit of 20 μm) was better than 0.3 eV. The binding energy was calibrated using the Fermi edge, acquired at the sample with energy step of 50 meV.

The substrate material is an Ag cylindrical crystal sector (diameter 9 mm), with axis oriented along the [11̅0] direction and cylinder radius *r* = 16 mm. The center of the curved surface is oriented along the (111) direction, leading to A-type steps at one side of the sample and to B-type steps at the opposite side, with vicinal angles α up to approximately 16°, which correspond roughly to the (559) and (221) surfaces, respectively (Figure 1a). When the sample is exposed to synchrotron light, the 20-μm spot size perpendicular to the surface illuminates an area at the desired vicinal angle, with a reduced spread of ±0.04°. This value matches the best surface misorientation value from flat single-crystal producing companies. The curved Ag(111) crystal was mounted on a sample holder provided with resistive heating and it was cleaned in the preparation chamber by cycles of sputtering parallel to the steps along the [11̅0] direction at an incident angle of 45° with Argon ions at 1 kV and *p* = 2 × 10^−6^ mbar and subsequent annealing to *T* = 450 °C. Cycles were repeated until sharp LEED spots were observed at the (111) part of the sample and the characteristic spot splitting occurred for the vicinal surfaces. The sample was then transferred to a near-ambient pressure photoemission chamber equipped with a SPECS 150NAP analyzer.

The flow of O_2_ into the analysis chamber was set to 5 mL/min using a mass flow controller (Brooks GF100C), and the pressure was regulated to 1 mbar using a manual valve connecting the analysis chamber to a roots pump. The pressure was measured with a capacitance manometer MKS Baratron 121AA installed in the analysis chamber. A mass spectrometer installed in the second pumping stage of the analyzer was used to monitor the gas near the sample. Oxygen gas was provided by Linde S.A. in a quality of 6.0. The impurities provided by the company do not specify any sulfur containing gas below the 10^−7^ level.

Peak fit analysis has been carried out for both O 1s and S 2p emissions with either Lorentzian and Doniach-Šunjic lineshapes^89^ accounting for the gas line and surface contributions, respectively. Convolution with a Gaussian is included to account for the instrument resolution. A Shirley background^90^ was used to account for secondary electron emissions from the corresponding core levels.

## Supporting Information Available

Choice of photon energy, impurity check, Ag-oxidation at room temperature, uptake experiments, Ag-oxidation above *T* = 100 °C, O 1s, Ag 3d and S 2p core levels at different vicinal angles, quantification of the S/O relation, O 1s and S 2p scans across the curved surface (α-scans), O 1s scan across the curved surface (α-scans) for two different curved crystals, and gas line energy shift (PDF)
Furthermore, a video is added that shows the curved sample setup during the synchrotron measurement (MP4)
