# Supplementary material for: Near-Ambient Pressure Oxidation of Silver in the Presence of Steps: Electrophilic Oxygen and Sulfur Impurities
Source: ACS Catal. 2024 Aug 12;14(17):12865–74. doi: 10.1021/acscatal.4c02985 (PMC11385363; doi:10.1021/acscatal.4c02985)
Supplement: Supplementary file 1 — cs4c02985_si_001.pdf [file cs4c02985_si_001.pdf]

Near ambient pressure oxidation of silver in the presence of steps: electrophilic oxygen and sulphur impurities

Supplementary information

Frederik Schiller,[*,†,‡] Khadiza Ali,[†,¶] Anna A. Makarova,[§] Sabine V. Auras,[†] Fernando García-Martínez,[†,‖] Alaa Mohammed Idris Bakhit,[†] Rodrigo Castrillo Bodero,[†] Ignacio J. Villar-García,[⊥,#] J. Enrique Ortega,[@,†,‡] and Virginia Pérez-Dieste[⊥]

† *Centro de Física de Materiales CSIC/UPV-EHU-Materials Physics Center, E-20018 San Sebastián, Spain*

‡ *Donostia International Physics Center, E-20018 San Sebastián, Spain*

¶*Department of Microtechnology and Nanoscience, Chalmers University of Technology, SE-41296 Göteborg, Sweden*

§*Physikalische Chemie, Institut für Chemie und Biochemie, Freie Universität Berlin, Arnimallee 22, 14195 Berlin, Germany*

‖*Deutsches Elektronen-Synchrotron DESY, Notkestraße 865, 22607 Hamburg, Germany*

⊥*ALBA Synchrotron Light Source, Cerdanyola del Vallès, 08290 Barcelona, Spain*

#*Departamento de Química y Bioquímica, Facultad de Farmacia, Universidad San Pablo-CEU, CEU Universities, 28668 Boadilla del Monte, Spain*

@ *Universidad del País Vasco, Dpto. Física Aplicada I, E-20018 San Sebastián, Spain*

E-mail: frederikmichael.schiller@ehu.es

This supplementary information file contains:

1. Choice of photon energy, impurity check
2. Ag oxidation at room temperature, uptake experiments
3. Ag oxidation above T = 100 °C, O 1s, Ag 3d and S 2p core levels at different vicinal angles
4. Quantification of the S:O relation
5. O 1s and S 2p scans across the curved surface (α-scans)
6. O 1s scan across the curved surface (α-scans) for two different curved crystals
7. Gas line energy shift

Choice of photon energy, impurity check

Photon energies have been selected to enhance high surface sensitivity and cross section for O 1s, as well as to optimize the transmission function of the SPECS Phoibos 150-NAP analyzer. The latter demands kinetic energies above 100 eV, i.e., photon energies of approx. hν = 640 eV. The O 1s peak at this photon energy range is conveniently found on a flat background, see Fig. S1(a). Nevertheless, one can also observe a strong overlap of Auger and core level peaks, such as the Ag 3d and the Ag Auger. This hν = 640 eV energy also allows checking for trace impurities like C, Cl, S or Si, around E_B=300 eV. At this range, we see that Ag and O Auger features overlap with the C 1s and the S 2p core-levels, respectively. By switching to a somewhat reduced photon energy of 620 eV we can single out the C 1s peak, as well as the Cl and S 2p levels. Si 2p and Ag 4s emissions appear at the same binding energy of approx. 100 eV. Nevertheless, Si 2p emissions are sharp while the Ag 4s emission is relatively broad, and it is clear that sharp features are not observed. The O 1s emission for hν = 620eV is located at kinetic energies of 80 eV and hence slightly below the optimal

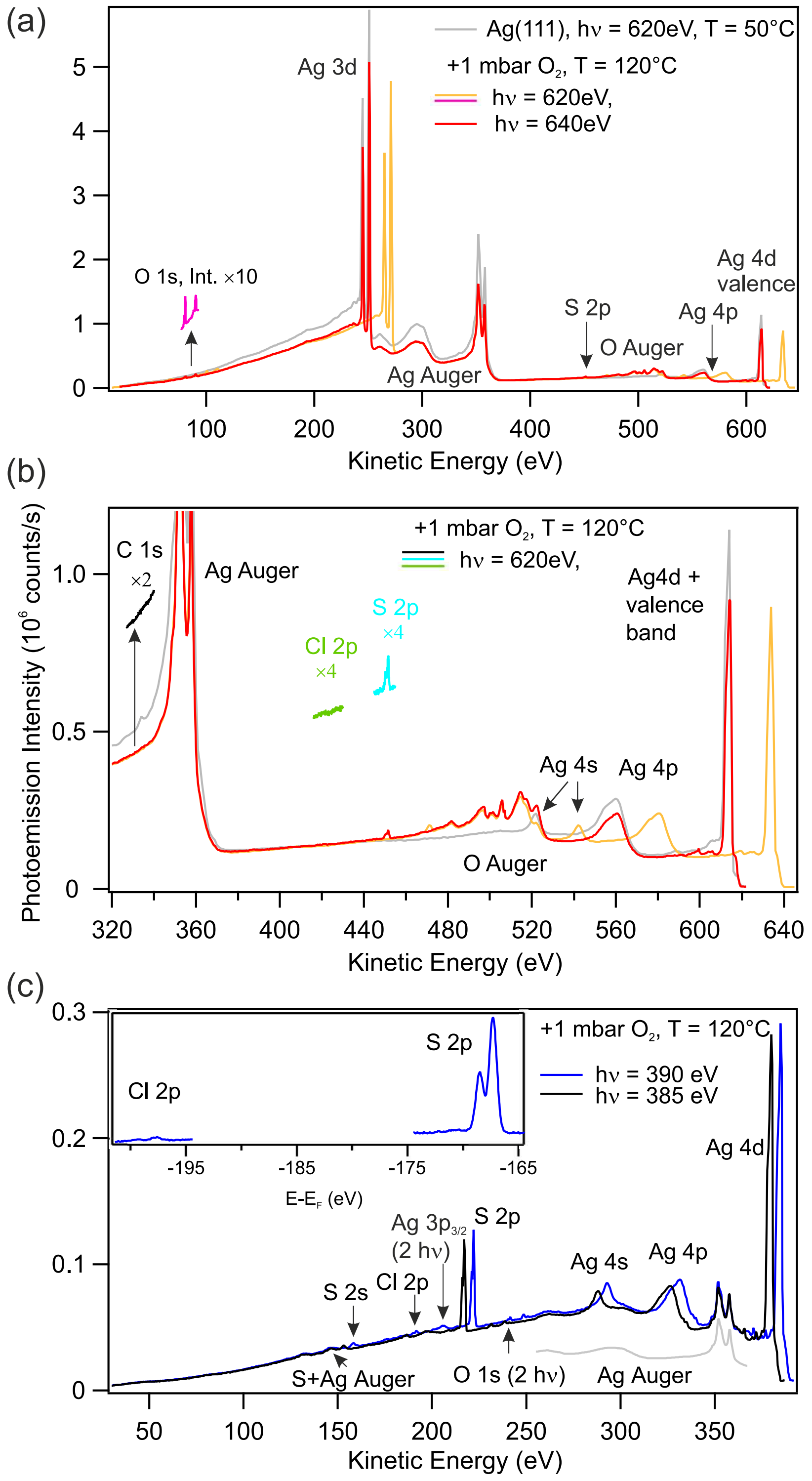

Figure S1: NAP-XPS survey spectra of Ag(111) after 2 hours exposure of 1 mbar O_2, at 120°C. (a) Full binding energy scan at hν = 640 eV and 620 eV. (b) Zoom-in at the high binding energy range to asses the presence of impurities. (c) Survey scans at hν = 390 eV and 385 eV.

100 eV. Yet, 620 eV still represents the best photon energy to measure all O 1s, Ag 3d, C 1s, Cl 2p, and S 2p core-levels without having to change the photon energy, see Fig. S1(b). In the two spectra shown in this figure, one cannot observe C 1s or Cl 2p. Another photon energy of hν = 390eV is used to verify the absence of C and Cl emissions, see Fig. S1(c). With such low photon energies, one observes a tiny Cl 2p feature. Other peaks in this panel correspond to Ag $M_3M_{4,5}N_{2,3}$ and S $L_{2,3}VV$ Auger excitations,[1] as well as to Ag 3p and O 1s core levels from second-order photon energies (2 hν). We conclude that at 1 mbar O_2 and 120°, we only observe sulphur and chlorine, with a S:Cl = 50:1 ratio.

Ag oxidation at room temperature, uptake experiments

For comparison, also the oxidation of Ag at room temperature was investigated. As already stated in the main text, at 1 mbar O_2 and room temperature, the C 1s emission reveals the presence of carbonates. In the bottom panel of Fig. S2, the evolution of the O 1s core level as a function of time is shown. Apart from the O_2 gas line doublet at higher binding energies, we observe carbonate emission at 530.5 eV and silver surface oxide at 528.1 eV. The latter changes its energy position towards 531 eV, i.e., the binding energy for bulk Ag oxide. The carbonate emission appears immediately after valve opening and decreases with time. A change of the sample position reveals that this decrease is partially beam-induced (not shown). Coming back to the original position, one can heat the sample to desorb the carbonate (top panel in Fig. S2 and Fig. 1 of the main text).

Ag oxidation above T = 100°C, O 1s, Ag 3d and S 2p core levels at different vicinal angles

In parallel to the O 1s and S 2p spectra shown in Fig. 3 of the main text, we also measured the Ag 3d, shown in Fig. S3. The Ag 3d core-level emission also evolves upon O_2 exposure.

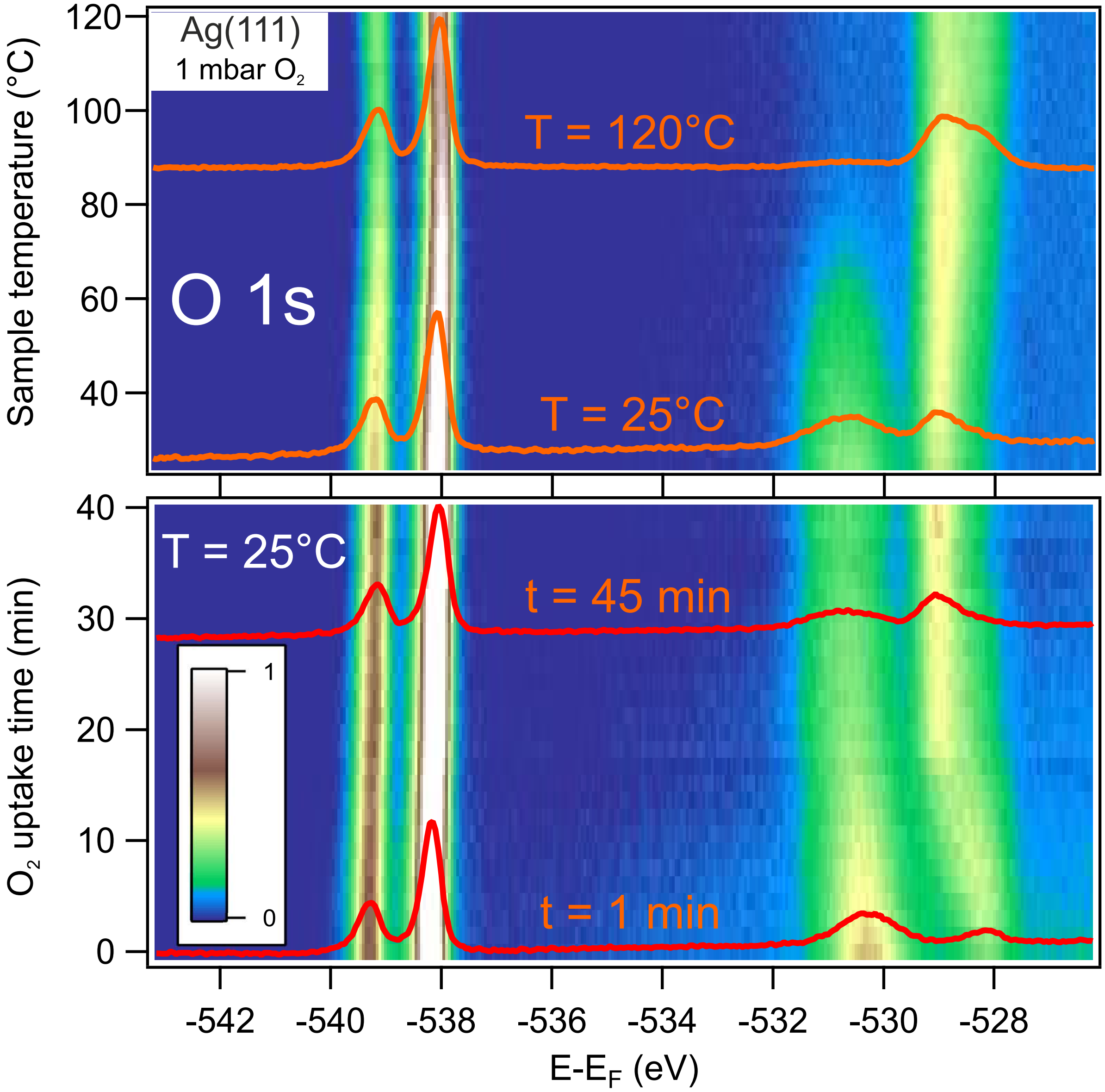

Figure S2: XPS O 1s spectra (bottom) during 1 mbar O_2 flow on Ag(111) ($t = 0$, $T =$ RT), h$\nu = 620$ eV, and (top) under continuous annealing at 3K/min. The latter image is identical to the one in the main article.

At the (111) surface there is a small change in the first 30 min, which correlates with the delayed formation of the bulk oxide. The bulk oxide forms much faster at the vicinal surfaces. The buildup of sulphur does not influence the Ag 3d emission.

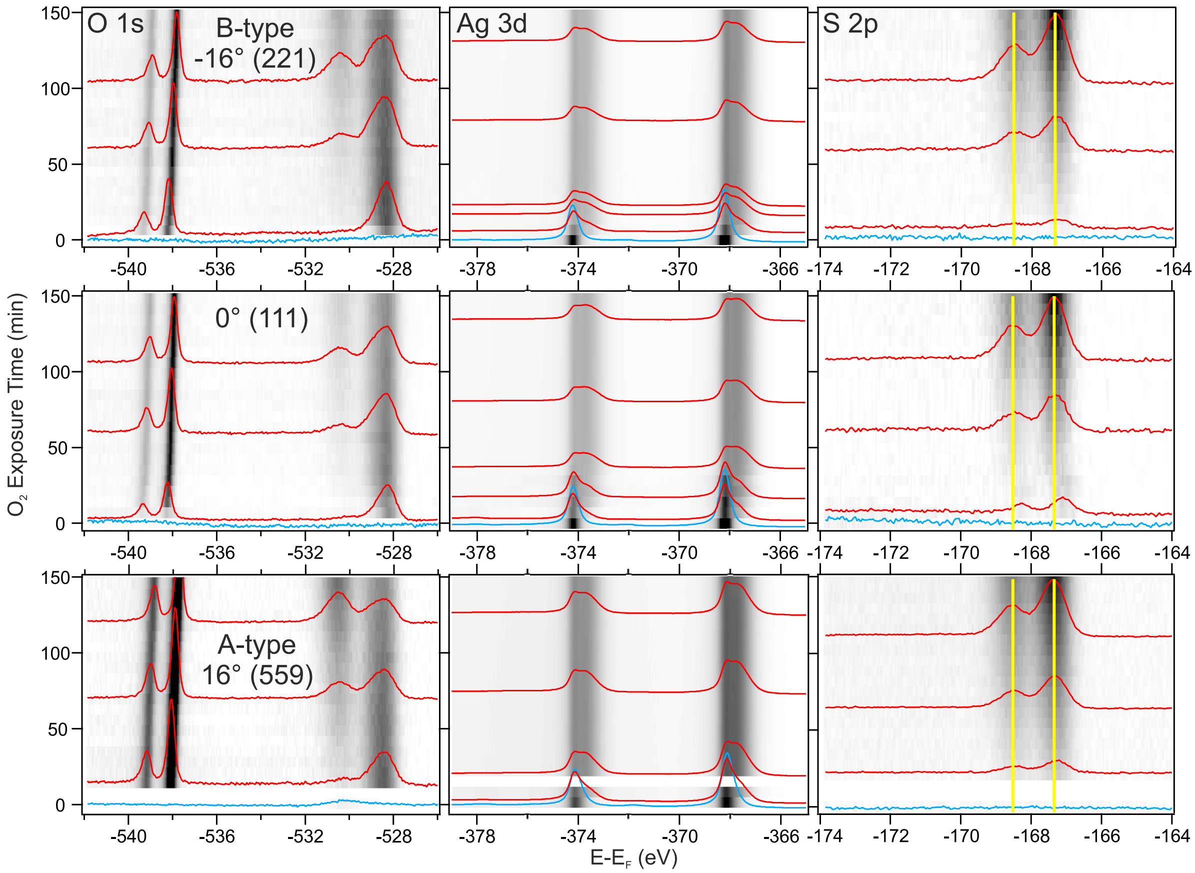

Figure S3: XPS O 1s, Ag 3d, and S 2p spectra during 1 mbar O_2 uptake at Ag(559), Ag(111), and Ag(221) positions (T = 120°C, hν = 620 eV). The O 1s and S 2p images are identical to those of the main text (but in gray scale).

Quantification of the S:O relation

The survey scan in Fig. S1(a) shows weak traces of O 1s emission, and relatively strong S 2p intensity. In fact the photoemission intensity drops drastically for low kinetic energies, and this mainly affects O 1s. This is caused by the analyzer transmission function and by the electron mean free path of the electrons in the gas atmosphere. For a correct S:O quantification, both effects need to be taken into account. Although the transmission function is defined by the analyzer tests,[2] and the photoelectron attenuation under NAP conditions can be reasonably estimated, we have evaluated the overall sensitivity/attenuation factor of the different core levels by measuring them at several photon energies, i.e., photoelectron kinetic energies, with and without gases. With this sensitivity factor, the S:O relation can

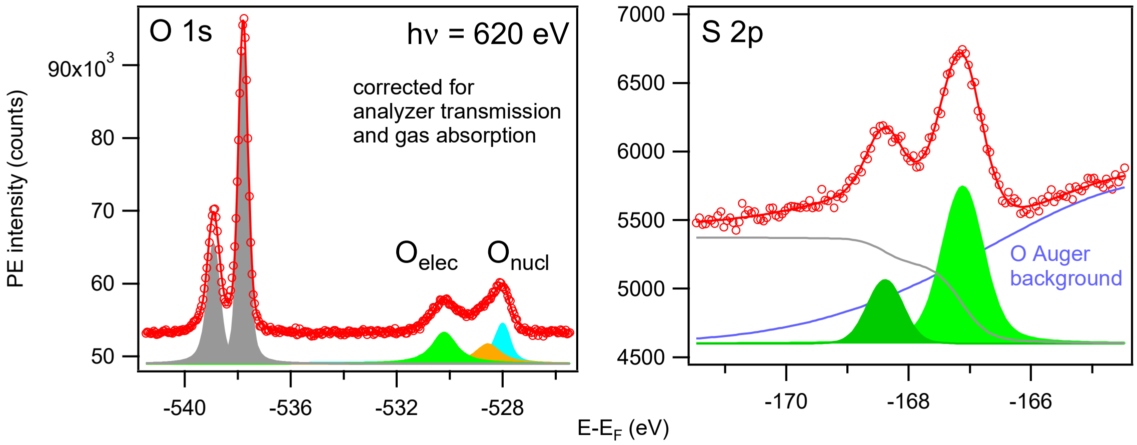

Figure S4: XPS O 1s and S 2p spectra during 1 mbar O_2 uptake on Ag(559) at T = 120 °C, with photon energy tuned to hν = 620 eV. Given the difference in kinetic energies for O 1s and S 2p, the O 1s intensity scale has been normalized to account for the different analyzer transmission function and photoelectron attenuation with respect to the S 2p core level. The O 1s and S 2p core levels were fitted to extract the intensities of the electrophilic O 1s and the S 2p emissions following the procedure explained in the main text.

be derived from the relative intensities obtained in the peak fits of the O 1s and the S 2p core levels in Fig. S4, and the photoemission cross section for the respective core level lines.[3] Note that only the electrophilic emission is considered for the quantification analysis. With such a procedure we get an estimation of S:O = 1:(3.8±0.6), very close to the suggested S:O = 1:4 composition of SO_4.

O 1s and S 2p scans over the curved surface

In Fig. S5 we examine O 1s (left) and S 2p (right) scans acquired in parallel over the curved surface (α-scans). Fig. S5(a) corresponds to the scan performed after 4 hours of O_2 dose at 170°C, corresponding to Fig. 6(b) of the main text, and Fig. S5(b) is a scan acquired immediately after O_2 dosing begins, with a substrate temperature of 120°C. Similar to the observations of Fig. 6 of the main text, one can again notice the strict correlation of the electrophilic O emission at 530.2 eV binding energy and the S 2p emission over the entire curved surface.

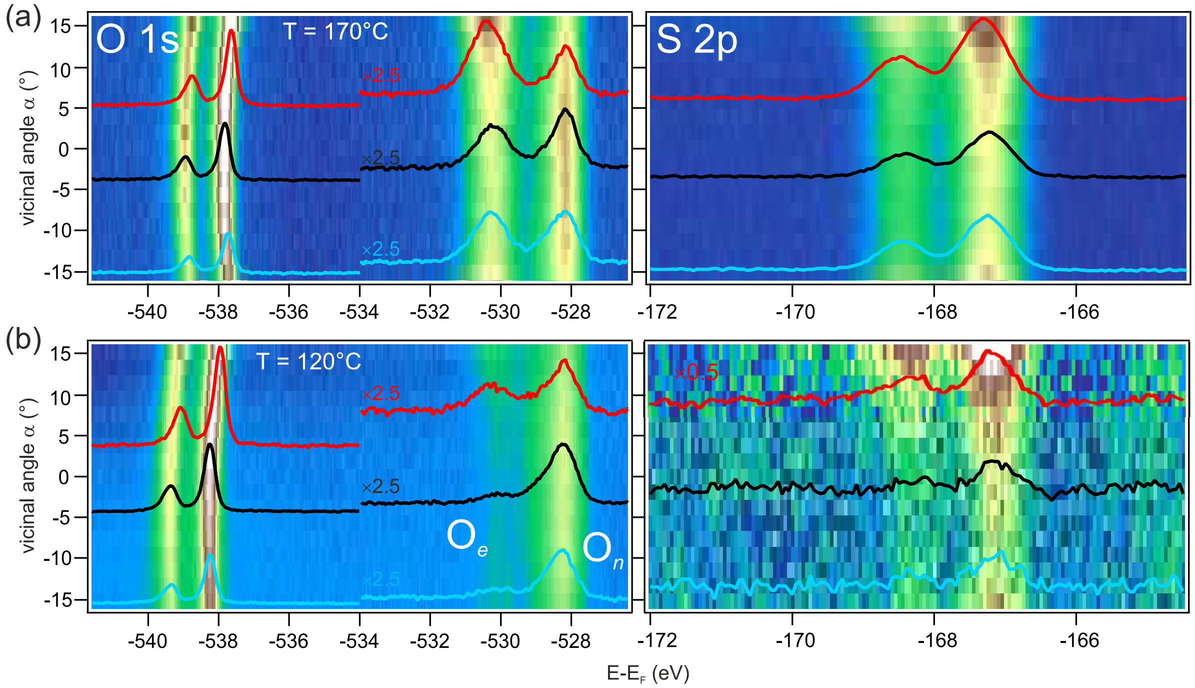

Figure S5: XPS scans of O 1s and S 2p core levels over the curved surface of the cylindrical crystal for two different preparations. (a) Clean sample exposed 4 hours to continuous O_2 dosing at 1 mbar, T = 170 °C. (b) Scan started immediately after dosing with continuous O_2 flow at 1 mbar, T = 120 °C

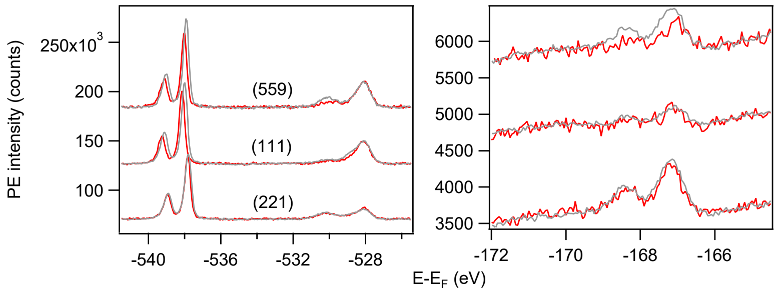

Figure S6: XPS scans of O 1s and S 2p core levels during the α-scan (red), which begins at the Ag(559) and ends at the Ag(221) sample edge, as compared to spectra acquired immediately after the α-scan is finished (gray). (O_2 flow at 1 mbar, T = 120 °C)

Figure S6 assesses the time evolution in one of the α-scans. For this purpose, three positions were re-measured at the end of the 17-points scan, namely the one at the starting point of the scan, Ag(559), the central position, Ag(111), and the last point, Ag(221). As stated in the main text, there is a continuous accumulation of SO_4 on the surface. The deviation is large when the delay time is the longest [30% intensity increase at Ag(559)],

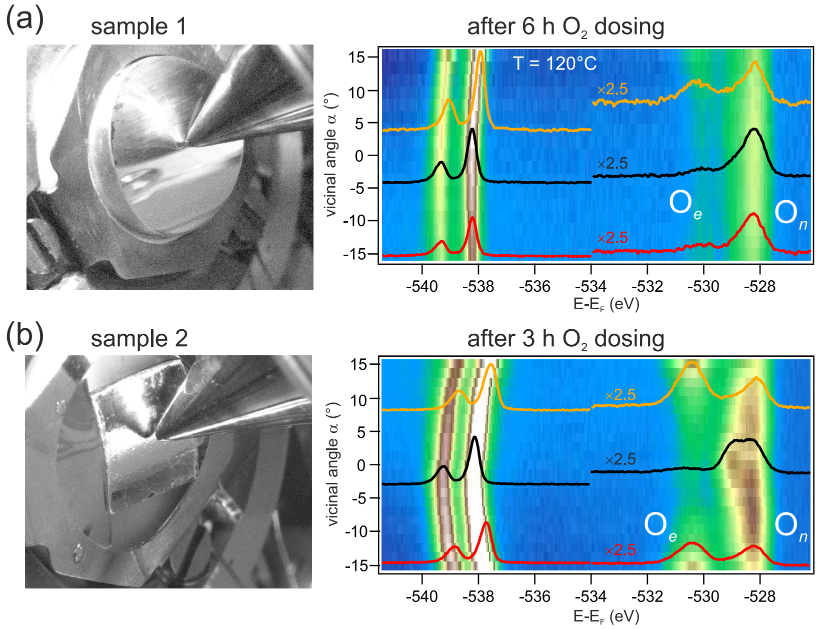

Figure S7: O 1s curvature scans for two different curved samples. At sample 1 accumulation of electrophilic O and S was more difficult to achieve and needed longer time (see also Fig. S5). Sample 2, on the other side, revealed a much faster increase of electrophilic O. Note, that in this experiment, the (111) central part was illuminated with the beam during the preceding 3 hours that leaded to a continuous increase in the nucleophilic bulk oxide due to the mentioned formation of atomic O or O_3 with the X-ray beam. This gave rise to the rather high amount of nucleophilic bulk oxide near (111) in comparison to sample 1 where the curvature α-scan was taken at non-illuminated positions. (O_2 flow at 1 mbar, T = 120 °C)

and it is no significant elsewhere, particularly at the last point in the scan, where the delay between the two curves is only a few minutes.

O 1s scan across the curved surface (α-scans) for two different curved crystals

One important issue is the origin of the sulphur. It could be either arise from the sample, the gas or from the surrounding vacuum conditions. In order to probe the sample origin, we used two different curved crystals but maintaining the gas and temperature conditions (O_2 flow at 1 mbar, T = 120 °C). The results of the two α-scans are shown in Fig. S7. One

clearly sees that accumulation of sulphur is much faster for sample 2 compared to sample 1. In the former case, sample 2, the accumulation time prior to the α-scan was three hours. In that time the (111) center was exposed to the beam and the oxygen flow. As stated in the main text, the X-rays together with the oxygen produce atomic O and/or O_3 that give rise to the formation of the (nucleophilic) bulk oxide near the illuminated spot (see Figure 3 of the main text). This causes a quite strong bulk oxide peak compared to the other sites at sample 2. Independently, the electrophilic O emission is quite strong, especially at the vicinal angles with an even higher electrophilic O amount compared to the nucleophilic O emission. Sample 1, on the other hand side, that was treated more often with sputtering/annealing cycles inside the vacuum, resulted to have less sulphur on the surface, even after long oxygen exposure times. Still, after 6 hours exposure plus the necessary time to measure the α-scan, the highest amount of electrophilic O is only half of the one of the nucleophilic surface oxide.

Gas line energy shift

The O_2 gas lines are very sensitive to small changes at the surface work function. The here observed changes can be traced to the SO_4 amount at the surface. In Fig. S8 we plot the gas line shift as a function of the electrophilic oxygen intensity for the three positions, Ag(221),

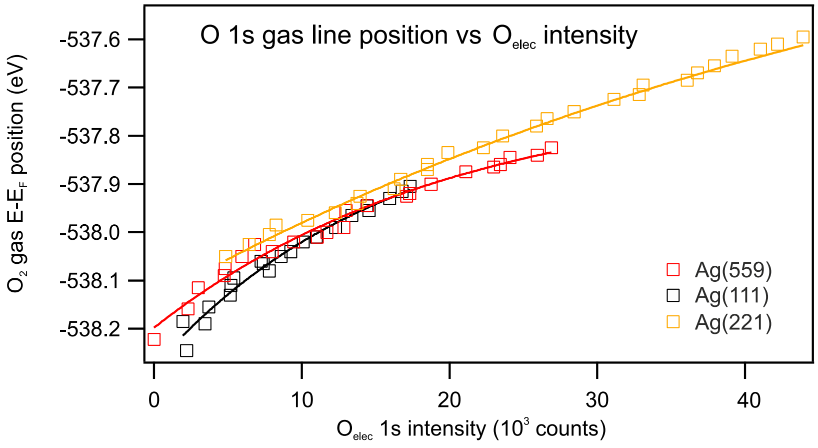

Figure S8: Binding energy shift of the O_2 gas line as a function of the O_{elec} intensity at (221), (111), and (559) positions of the uptake spectra shown in Fig. 3 of the main text.

Ag(111) and Ag(559) during the 1 mbar O_2 dosage shown in Fig. 4 of the main text. There is a small offset between the curves but mainly it follows a nearly linear trend.

References

1. Coad, J. P.; Rivière, J. C. Origin of fine structure in the Auger spectrum of sulphur on a nickel surface. *Proceedings of the Royal Society of London. A. Mathematical and Physical Sciences* **1972**, *331*, 403–415.

2. Specs G.m.b.H., Specs 150NAP transmission function. Accessed April 24, 2024. https://www.specs-group.com/fileadmin/user_upload/products/technical-note/TNote-PHOIBOS_150_NAP_Calculated_Transmission_function.pdf.

3. Yeh, J. J.; Lindau, I. Atomic subshell photoionization cross section and asymmetry parameters: $1 \leq Z \leq 103$. *At. D. and Nucl. Data Tables* **1985**, *32*, 1.
